# Automatic lesion segmentation using atrous convolutional deep neural networks in dermoscopic skin cancer images

**DOI:** 10.1186/s12880-022-00829-y

**Published:** 2022-05-29

**Authors:** Ranpreet Kaur, Hamid GholamHosseini, Roopak Sinha, Maria Lindén

**Affiliations:** 1 School of Engineering, Computer, and Mathematical Sciences, Auckland University of Technology, 55 Wellesley street, 1010 Auckland, New Zealand; 2 School of Innovation Design and Engineering, Mälardalen University, Västerås, Sweden

**Keywords:** Skin cancer, Lesion segmentation, CNN, Deep learning

## Abstract

**Background:**

Melanoma is the most dangerous and aggressive form among skin cancers, exhibiting a high mortality rate worldwide. Biopsy and histopathological analysis are standard procedures for skin cancer detection and prevention in clinical settings. A significant step in the diagnosis process is the deep understanding of the patterns, size, color, and structure of lesions based on images obtained through dermatoscopes for the infected area. However, the manual segmentation of the lesion region is time-consuming because the lesion evolves and changes its shape over time, making its prediction challenging. Moreover, it is challenging to predict melanoma at the initial stage as it closely resembles other skin cancer types that are not malignant as melanoma; thus, automatic segmentation techniques are required to design a computer-aided system for accurate and timely detection.

**Methods:**

As deep learning approaches have gained significant attention in recent years due to their remarkable performance, therefore, in this work, we proposed a novel design of a convolutional neural network (CNN) framework based on atrous convolutions for automatic lesion segmentation. This architecture is built based on the concept of atrous/dilated convolutions which are effective for semantic segmentation. A deep neural network is designed from scratch employing several building blocks consisting of convolutional, batch normalization, leakyReLU layer, and fine-tuned hyperparameters contributing altogether towards higher performance.

**Conclusion:**

The network was tested on three benchmark datasets provided by International Skin Imaging Collaboration (ISIC), i.e., ISIC 2016, ISIC 2017, and ISIC 2018. The experimental results showed that the proposed network achieved an average Jaccard index of 90.4% on ISIC 2016, 81.8% on ISIC 2017, and 89.1% on ISIC 2018 datasets, respectively which is recorded as higher than the top three winners of the ISIC challenge and other state-of-the-art methods. Also, the model successfully extracts lesions from the whole image in one pass in less time, requiring no pre-processing step. The conclusions yielded that network is accurate in performing lesion segmentation on adopted datasets.

## Introduction

Skin cancer is caused by the growth of cancerous cells that proliferate in an abnormal and uncontrolled manner in the topmost layer of the skin called the epidermis. The primary reason for the occurrence of skin cancer is direct exposure to ultraviolet sun rays for longer hours producing a pigment known as melanin in the upper skin layer [1]. Moreover, fair complexion, sunburn, genetic history, and weak immune system are other risk factors that contribute to the formation of skin cancer [2]. There are different types of skin cancer, such as squamous cell carcinoma, basal cell carcinoma, and melanoma [3], where melanoma is the most aggressive form of cancer comparatively. According to the statistics reported by the American skin cancer society [4], melanoma is the 19th most commonly found problem worldwide, and 100,350 new cases of melanoma were anticipated in the USA, 16221 in Australia, and 2500 in New Zealand in 2020 [5]. In terms of pricing, it has been estimated that the cost of treating skin cancer is 3.3 billion per year [6, 7]; thus, it is the most expensive procedure for the health systems.

**Fig. 1:**
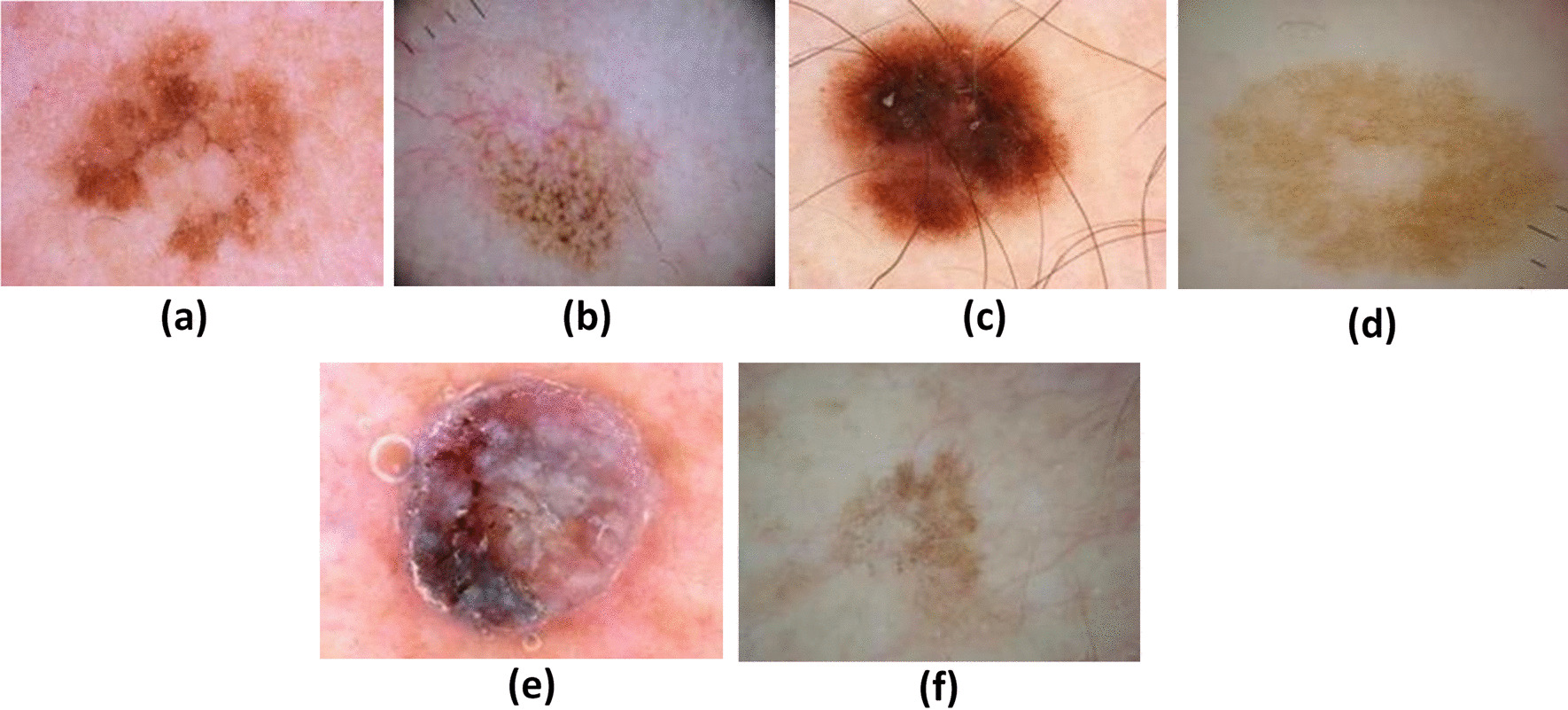
Examples of noise artifacts. **a** Irregular boundaries, **b** blood vessels, **c** hairlines, **d** color illumination, **e** bubbles, **f** low contrast

Moreover, melanoma has become a critical public health concern for clinicians and researchers who emphasize reducing the mortality rate with early diagnosis. Detecting melanoma early can increase the survival rate. In clinical settings, trained specialists such as dermatologists commonly diagnose melanoma from dermoscopic lesion images based on Asymmetry, Border, Color, Diameter, and Evolution (ABCDE) [8] characteristics which is a very time-consuming process. Other methods used by dermatologists for performing visual examination are biopsy and histopathological analysis. The major problem in these traditional diagnostic procedures is time, high cost, and variation inaccuracy. Thus, computer-aided design (CAD) systems are widely adopted for the timely detection of melanoma, where cancer image segmentation is the most crucial process in CAD for the detailed analysis of lesion structure. The heterogeneous appearance of the lesion area in terms of color, size, shape, location, and texture makes the segmentation task very challenging, as shown in image samples of Fig. 1. Therefore, there is a need for an automatic segmentation approach to assist dermatologists in understanding the nature and pattern of the lesion area. Also, this method is significant for automatically generating the ground truth images which were previously annotated manually by dermatologists.

Recent advances in artificial intelligence, particularly deep learning, offered numerous automatic and accurate identification advantages. Therefore, for extracting accurate lesion patterns in skin cancer images, a novel deep neural framework is proposed that proved to be a suitable segmentation approach based on the obtained results for the given datasets. We also embedded the atrous convolutions [9] in the feature extraction layers of the network, which improved performance and maintained the spatial resolution of the segmented images. In addition to this, the design of a deeper network allows the extraction of shallow features and in-depth features for performing more accurate segmentation. The designed network was evaluated on a benchmark dataset collected from the last three years (2016, 2017 and 2018) by ISIC [10–13].

Our contributions are listed as: (a) a new design of the CNN network organized into five building blocks for extracting low-level features to high-level features to allow auto segmentation on the whole image rather than processing a patch or subset, (b) incorporating an atrous dilation rate in each convolution feature layer to capture lesion and image at different scales, (c) a careful placement of many leakyReLU activation functions in each block instead of standard ReLU because the former has a small slope for negative values that makes the network learns faster and is more balanced.

This framework is designed to meet two main challenges: (a) lesion segmentation with high performance on images containing irregularities and non-uniform borders, (b) achieving low inference time, making the network faster.

## Background

Researchers have made several attempts to develop image segmentation techniques for accurate lesion extraction. In the literature, segmentation approaches have been categorized into six categories: Edge-based methods [14], Thresholding-based methods [15], Clustering [16], Active contour [17], and Supervised approaches such as ANN [18]. Deep learning has been widely used for classification and object detection, where the idea of applying it to semantic segmentation has been an active area of research over the past few years.

Deep neural networks are effectively applied in semantic segmentation tasks to label each pixel with a class of object or non-object region. Some of the most popular CNNs proposed by the researchers to attempt segmentation task were fully convolutional neural network (FCN) [19], deconvolution networks (DeconvNet) [20] as an extension to FCN networks, SegNet [21], and UNet [22]. They are purely data-dependent, so their performance differs depending on the problem domain. A few limitations exist in the design of these networks, such as FCN uses pooling layers that reduce resolution and discards essential image information. However, semantic segmentation requires exact knowledge of class maps and needs to preserve ’where’ information. Similarly, the encoder-decoder networks (SegNet and UNet) are efficient in maintaining output image resolution for some problem domains; however, networks become heavy and take high execution time due to many sampling and downsampling layers. In contrast, the concept of atrous convolutions introduced by Chen et al. [23] allows direct control of the resolution to preserve feature map information computed in the deep convolutional layers but have a high computation time due to its large network design.

Many studies proposed an extended version of these networks to perform segmentation of melanoma. For example, Kawahara et al. [24] extended VGG16 architecture to perform lesion segmentation by eliminating its fully connected layers and resizing the final feature map so that it matches with the output size. Bi et al. [25] proposed an FCN based ResNet model that learns the visual features of the lesion corresponding to each class based on their probability. This network is significantly different from other networks as it segments images according to their category and learns more in-depth features. Another work of Yu et al. [26] presented a very deep residual network having 50 layers to calculate rich and more significant features for accurate recognition. Residual learning was applied to prevent overfitting and degradation problems of the convolutional neural networks. For lesion segmentation using a convolutional network, Al-Masni et al. [27] presented the full resolution convolutional network (FRCN) without any preprocessing. The VGG16 layers inspired the network by removing their subsampling layers to preserve pixels in their full resolution. According to work presented in [28] a convolutional multistage segmentation network is highly efficient in obtaining lesions from skin cancer images. In this, multiple stages of the network integrated outputs from different blocks combined with other steps. To create a fine segmentation mask, the network used pixel classification. In a study by Hassan et al. [29] dermoscopic skin network (DSNet) was used to segment lesions. A deeper view of the feature maps was obtained by using depth-wise separable convolutions instead of standard convolutions. A similar type of lesion segmentation work can be seen in the [30–33] that given different approaches for lesion segmentation either inspired by existing networks or extending them using transfer learning.

Recent developments have heightened the need for a melanoma detection system, and considerable literature has grown up around the theme of lesion segmentation. It has been analyzed that the most popular network’s choices for semantic segmentation are U-Net, FCN, and SegNet, containing sampling and upsampling layers to maintain the spatial resolution of the output. The major problem in these networks is that they suffer from a low spatial resolution output due to the repetitive use of max-pooling and striding at consecutive layers. Moreover, the optimized, extended, and customized frameworks proposed in the literature for lesion segmentation tasks still exhibit difficulties such as low performance or high execution time due to complex architectures. These networks have not given satisfactory performance on the adopted skin cancer dataset. Hence, there is probably room for further improvement in performance by designing a new architecture. In the proposed work, we employ atrous convolutions that effectively maintain the segmented image’s resolution. The network is designed from scratch by organizing different layers of the network, fine-tuning hyperparameters, and using a suitable loss function. This network has the advantage of being a small network with low execution time, minimal learning parameters, and high performance.

## Methods and materials

This section explains the preparation of the dataset used for training, validation, and test, architectural details of the atrous convolution-based deep neural network, and discussion of metrics used for performance evaluation.

### Datasets

In this study, three benchmark datasets were acquired from open-access dermatology repositories, ISIC archive [10–13], containing dermoscopic images of different skin cancer types such as Basal cell carcinoma (BCC), Melanoma, Squamous, and Nevus, including their ground truths which were used for training, validation, and testing purposes. The ISIC 2016 dataset contains 900 training and 379 testing images, ISIC 2017 has 2000 training, 150 validation, 366 testing images, and ISIC 2018 consist of 2594 training and 100 validation. The ISIC 2016 challenge has not provided external validation data; hence, the training set was divided in the ratio of 7:3 into training (630 images) and validation sets (270 images). Similarly, in ISIC 2018 challenge, the ground truth for test data was not provided; hence, the 30% of training data was used for testing purposes. The details of data available based on three years divided into different sets are given in Table 1.

**Table 1:** Details of the ISIC challenge data

Dataset name	Training	Validation	Test
ISIC 2016	630	270	379
ISIC 2017	2000	150	366
ISIC 2018	1816	100	780
Total	4446	520	1525

The proposed approach targets dermoscopic images, which were produced by dermatoscopes. Available images are 8-bit with size ranges from $540\times 722$ to $4499\times 6748$ pixels. These images cannot be used directly for the network’s training due to their large size; thus, images are downsized to $192\times 256\times 3$ dimensions using the nearest-neighbor interpolation technique. The principle idea used in this resizing method is to have an original image as a reference image based on which a new rescaled image is constructed. The constructed image results in a smaller size maintaining the aspect ratio and resolution as the original image. Different image sizes were taken into consideration during experiments, such as $224\times 224$, $227\times 227$, $256\times 297$ but $192\times 256\times 3$ had given the best performance for the proposed network. Data augmentation with rotation at an angle between $[15^{\circ },20^{\circ }]$, scaling, and translation with factors $[-6,5]$ is applied to enlarge the training dataset and to overcome the problem of underfitting or overfitting that occurs in neural networks owing to the insufficient availability of data.

### Proposed DilatedSkinNet architecture

**Fig. 2:**
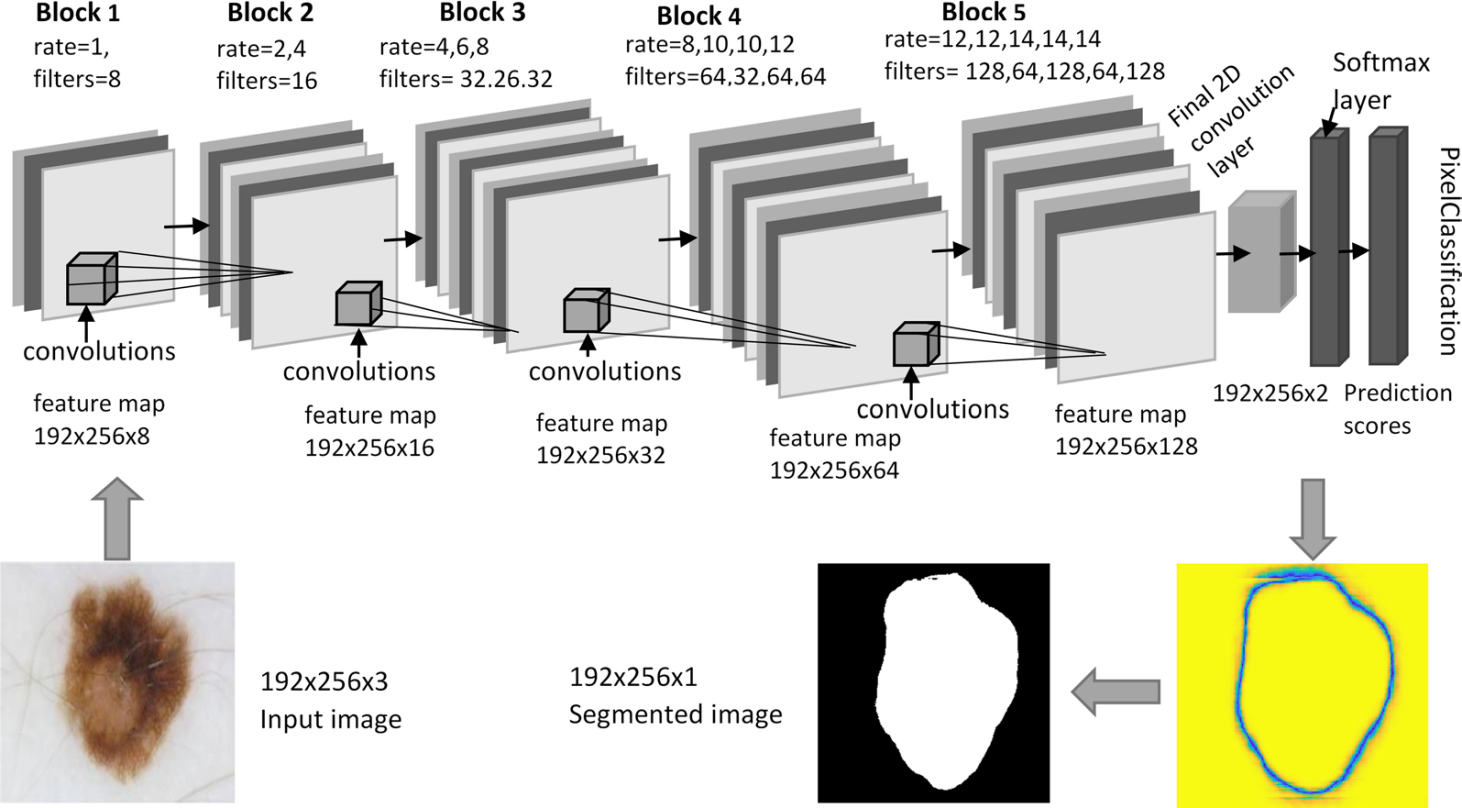
DilatedSkinNet architecture. An overall layered structure of the proposed method

With the invention of atrous convolutions in CNN architecture by Chen et al. [9, 23] to achieve a wider field of view, research on the development of dilated CNN networks continued for different applications, and a high execution time is one of the significant challenges required to achieve. In this paper, we designed an end-to-end trainable deep neural network architecture having 16 convolutional layers with different dilation factors, as shown in Fig. 2. The structure of DilatedSkinNet is interpreted in two primary steps: feature extraction and pixel classification. Using multiple scaling rates, the network used atrous convolutions to enlarge the filter’s view. The pooling operation used in other semantic networks reduces the dimensional size of output feature vectors and the loss of information. In contrast, dilated convolutions expand the receptive field’s view to obtain in-depth information without using pooling operation and are suitable for maintaining the spatial resolution of the segmented image. This section discusses the working of networks divided among various layers such as feature extraction, use of atrous convolutions, normalization, activation function, and classification.

#### Feature extraction

The convolutional layer in the network is responsible for performing the feature calculation process. The initial image is divided into multiple sub-blocks based on a certain size and those sub-blocks compute features of the input image. The feature calculation is a process where a small matrix called kernel or filter slides over an image and transforms the image pixel’s values as:

1 $$\begin{aligned} {\text {C(I,K)}}_{\mathrm{x,y}} =\sum _{{\mathrm{i}}=1}^{{\mathrm{m}}_{\mathrm{h}}}\sum _{{\mathrm{j}}=1}^{{\mathrm{m}}_{\mathrm{w}}}\sum _{{\mathrm{k}}=1}^{{\mathrm{m}}_{\mathrm{c}}} {\text {I}}_{\mathrm{x+i-1, y+j-1,k}}*{\text {K}}_{\mathrm{i,j}} \end{aligned}$$

Here $m_{h}$ is the height, $m_{w}$ is width, and $m_{c}$ is the number of channels of an input image I. The number of channels of a kernel needs to be the same as the channels of an input image. The other parameters that we set artificially in the convolution layer are stride and padding. However, the dilation rate is set in each convolutional layer, so the stride and padding are zero in the network. Multiple filters are used in each convolutional layer to generate feature maps (see Table 2). The advantage of using multiple filters is that each filter convolves over the whole image separately to calculate many different features and produces the rich feature map used by the next layers.

#### Atrous/dilation CNN model

The use of atrous convolutions with different dilation rates at each convolutional layer helps extract a rich feature map. It allows increasing the view of the filter’s field (the space of the input vector that a layer can see) to assimilate a larger context. Therefore, it provides an efficient methodology to determine the best trade-off between correct localization and context absorption without increasing the computational parameters.

In the convolutional layers, the convolution operation is performed by sliding a template over an image for extracting features. We used atrous convolutions instead of general convolutions, which are decisive for extracting more contextual information. The standard convolutional operation is described in (2) with the dilation rate always ‘1’.

2 $$\begin{aligned} {\text {C[i]}}\;=\;\sum _{{\mathrm{s}}=1}^{\mathrm{S}}{\text {I[i + s]}}\;*\;{\text {K[i]}} \end{aligned}$$

whereas, (3) describes the atrous convolution operation when the dilation rate is more than ‘1’.

3 $$\begin{aligned} {\text {C[i]}}\;=\;\sum _{{\mathrm{s}}=1}^{\mathrm{S}}{\text {I[i + s.r]}}\;*\;{\text {K[i]}},\; \; {\text {r}}\ge {2} \end{aligned}$$

**Fig. 3:**
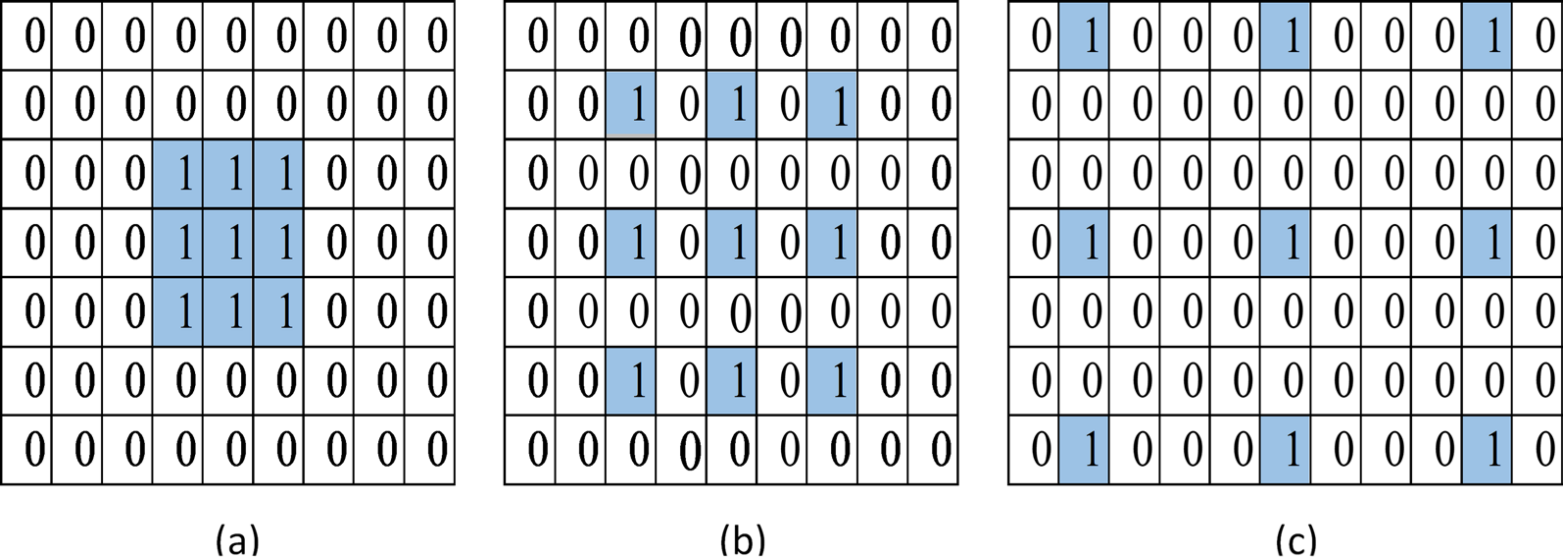
Standard convolution. **a** Rate = 1, Atrous convolution, **b** rate = 2, **c** rate = 4

We used two kernel sizes, $3\times 3$ and $1\times 1$, in the atrous convolutional layers with different dilation rates at each layer to overcome the ‘gridding effect’ that occurs due to the use of the same dilation rate. The dilation factor increased at a rate of ‘2’ at each successive 2-dimensional convolutional layer. Figure 3 represents the impact of using dilation rate ‘2’, ‘2’ and ‘4’ on filter’s view over an input image.

#### Data normalization

The batch normalization is used to speed up the process of network learning using internal normalization values. The word ‘batch’ refers to the group or set of data processed at a time, where mini-batch size is a variation of the gradient descent algorithm that splits the training dataset into small batches to calculate network error and update network coefficients. This layer in the network is used to normalize each input according to the mini-batch size. The batch normalization layer performs a series of operations to normalize the data. Firstly, the standardization process converts the batch of input data so that their mean is zero and the standard deviation is one. The mean $(\mu )$ is computed using (4) by summing up all inputs $(x_{i})$ of the batch (B) and divided by the total number of inputs (n). The resultant vector contains each input sample’s mean value. The variance $(\sigma ^{2}_{B})$ (5) is obtained by squaring the standard deviation of the input. It is computed by taking the square of each input sample $(x_{i})$ in the current batch (B) subtracted from the mean $(\mu _{B})$.

4 $$\begin{aligned}{\text {mean}}, \mu _{\mathrm{B}}= \frac{1}{{\text {N}}}\sum _{{\mathrm{i}}=1}^{\mathrm{N}} {\text {x}}_{\mathrm{i}} \end{aligned}$$

5 $$\begin{aligned}{\text {variance}}, \sigma ^{2}_{\mathrm{B}}= \frac{1}{{\text {N}}}\sum _{{\mathrm{i}}=1}^{\mathrm{N}} ({\text {x}}_{\mathrm{i}}-\mu _{\mathrm{B}})^2 \end{aligned}$$

In the following operation (6), the mean value of the current batch is subtracted from each input instance and divided by the square root of addition between standard deviation and smoothing term $\epsilon$. This term is set as ‘0.00005’ to avoid division by a zero number in the calculations.

6 $$\begin{aligned}\hat{{\text {x}}_{\mathrm{i}}}=\frac{{\text {x}}_{\mathrm{i}}-\mu _{\mathrm{B}}}{\sqrt{\sigma ^{2}_{\mathrm{B}}+\epsilon }} \end{aligned}$$

Finally, the output of the batch normalization process is obtained by re-scaling $\gamma$ and offsetting $\beta$ of the input values using (7). These two parameters were learned during the training process and optimized to ensure accurate normalization.

7 $$\begin{aligned} {\text {BN}}_{\gamma \beta }({\text {x}}_{\mathrm{i}}) \quad {\text {or}} \quad {\text {y}}_{\mathrm{i}}=\gamma \hat{{\text {x}}_{\mathrm{i}}}+\beta \end{aligned}$$

The batch normalization is generally placed between the convolutional and ReLU layers to stabilize the learning process and reduce the number of epochs. After each convolutional layer, the batch normalization is to reduce the internal covariate shift, which significantly improves the network’s learning efficiency.

#### Activation function

The activation function used in the network is leakyReLU, which applies the threshold function to each element in the input and multiplies all negative values by a fixed scalar ‘*a*’. This layer passes the output element as the input to the next layer directly if it is positive; otherwise, it outputs to a value multiplied by ‘*a*’ as given:

8 $$\begin{aligned} {\text {leakyReLU}}= {\left\{ \begin{array}{ll} {\text {a*x}},\; {\text {x}}<0, \\ {\text {x}},\; {\text {x}}\ge {0} \end{array}\right. } \end{aligned}$$

In the proposed CNN, the scalar ‘*a*’ value is set as ‘0.1’. The activation function is responsible for transforming the summed weighted input from the node into the node’s activation.

#### Classification

In the second part of the proposed network, instead of fully connected layers, a $1\times 1$ convolutional layer was created to flatten the feature maps and minimize the number of channels. Then, the SoftMax layer was used, accepting the sparse feature sets as input for classifying each pixel of the image into two defined classes, i.e., lesion and background. The SoftMax function normalizes the weighted sum feature values to probability scores between 0 and 1. The output of this layer was the probability of each pixel mapped to each class. The last layer of the network was the pixel classification layer that produces a categorical label (background or lesion) for each pixel based on the probability score generated by the SoftMax layer. This layer also uses a loss function to calculate the network’s prediction error rate. Over several iterations, the network’s training is repeated to minimize the loss function’s value. The structural details of the network illustrating a design of layers, kernel size, number of filters, and dilation rate are explained in Table 2.

**Table 2:** Architecture details of the DilatedSkinNet

Block	Layer	Kernel size,	Atrous dilation	Output size
		Feature maps	Rate	
Input layer	$192\times 256\times 3$	–	–	$192\times 256\times 3$
Block 1	Conv1	$3\times 3$, 8	1	$192\times 256\times 8$
Block 2	Conv2_1	$3\times 3$, 16	2	$192\times 256\times 16$
	Conv2_2	$3\times 3$, 16	4	$192\times 256\times 16$
Block 3	Conv3_1	$3\times 3$, 32	4	$192\times 256\times 32$
	Conv3_2	$1\times 1$, 16	6	$192\times 256\times 16$
	Conv3_3	$3\times 3$, 32	8	$192\times 256\times 32$
Block 4	Conv4_1	$3\times 3$, 64	8	$192\times 256\times 64$
	Conv4_2	$1\times 1$, 32	10	$192\times 256\times 32$
	Conv4_3	$3\times 3$, 64	10	$192\times 256\times 64$
	Conv4_4	$3\times 3$, 64	12	$192\times 256\times 64$
Block 5	Conv5_1	$3\times 3$, 128	12	$192\times 256\times 128$
	Conv5_2	$1\times 1$, 64	12	$192\times 256\times 64$
	Conv5_3	$3\times 3$, 128	14	$192\times 256\times 128$
	Conv5_4	$1\times 1$, 64	14	$192\times 256\times 64$
	Conv5_5	$3\times 3$, 128	14	$192\times 256\times 128$
	final_Conv	$1\times 1$, 2	–	$192\times 256\times 2$
	Softmax	–	–	$192\times 256\times 2$
OutPutMap	Pixel classification	Cross entropy	Loss function	$192\times 256\times 2$

#### Model training

The network is trained and optimised depending upon the loss function that measures the error between the prediction score P and target T. In this paper, the weighted cross-entropy loss [34] function was employed to measure the error as:

9 $$\begin{aligned} {\text {Loss}}=\frac{1}{{\text {N}}}\sum _{{\mathrm{i}}=1}^{\mathrm{K}}\sum _{{\mathrm{n}}=1}^{\mathrm{N}} {\text {w}}_{\mathrm{i}} {\text {T}}_{{\mathrm{n}}_{\mathrm{i}}} \log ({\text {P}}_{n_i}) \end{aligned}$$

Here, N is the number of observations, K is the number of classes, and w is a vector of weights determined by the network for each class. The stochastic gradient descent algorithm is used to update the network weights and biases to reduce the loss value by applying small changes in the direction of optimization.

10 $$\begin{aligned} \theta _{{\mathrm{i}}+1}=\theta _{\mathrm{i}}-\alpha \bigtriangledown {\text {L}}(\theta _{{\mathrm{i}}}) \end{aligned}$$

Here i is the number of iterations, $\alpha >0$ is the learning parameter (set as ‘0.01’), $\theta$ is a parameter vector, and $\bigtriangledown L(\theta _{i})$ is the gradient of the loss function. The algorithm evaluates the gradient at each iteration and updates parameters over a mini-batch set. The larger weight values can cause a network to be stuck into the local minima; thus momentum term is added in the gradient descent algorithm to reduce the oscillations as given in (11). The values of these hyperparameters set for the network’s training are shown in Table 3.

11 $$\begin{aligned} \theta _{{\mathrm{i}}+1}=\theta _{{\mathrm{i}}}-\alpha \bigtriangledown {\text {L}}(\theta _{{\mathrm{i}}})+\gamma (\theta _{{\mathrm{i}}}-\theta _{{\mathrm{i}}+1}) \end{aligned}$$

**Table 3:** Optimised hyperparameters during training

Parameter	Values
Input Image size	$192\times 256\times 3$
Batch size	16
Learning parameter, $\alpha$	0.01
L2Regularization	0.005
Momentum, $\gamma$	0.9
Loss function, $E(\theta )$	Weighted cross entropy loss
Optimiser	SGDM

### Evaluation metrics

The performance of the proposed DilatedSkinNet is evaluated quantitatively using performance metrics such as accuracy (ACC), Jaccard index (JAC), and Dice-coefficient (DICE). The value of these parameters was calculated for the test dataset and is expected to be higher for good segmentation results. The ACC parameter indicates the number of corrected pixels identified over the total number of pixels. A statistical measure to determine the similarity ratio between the ground truth and predicted label is known as the JAC index. The DICE computes the boundary contour matching index between the predicted and accurate segmentation in the ground truth.

12 $$\begin{aligned}{\text {ACC}} ={\frac{{\text {TP + TN}}}{{\mathrm{TP + TN + FP + FN}}}} \end{aligned}$$

13 $$\begin{aligned} {\text {JAC}}={\frac{{\text {TP}}}{{\mathrm{TP + FP + FN}}}} \end{aligned}$$

14 $$\begin{aligned} {\text {DICE}}={\frac{2*{\text {TP}}}{2*{\text {TP + FP + FN}}} } \end{aligned}$$

The parameters TP, TN, FP, and FN, denotes the true positives, true negatives, false positives, and false negatives, respectively, which are elements of the confusion matrix. TP represents those pixels that are segmented correctly, whereas incorrectly segmented pixels are considered FN. On the other hand, non-lesion pixels, if classified correctly then considered TN; otherwise, FP.

## Results and discussion

The proposed network is trained for three years’ datasets (ISIC 2016–2018) separately, having 4446 training images, 520 validation, and 1525 test images. The proposed network is implemented in Matlab 2020a with GeForce GTX 1080 Ti hardware configuration with a computation capacity ‘7.5’. To show the impact of using data augmentation, atrous convolutions, leakyReLU activation function, and use of sigmoid layer on the performance of the model are displayed in Table 4. It illustrated that the proposed model with augmentation, atrous convolutions, leakyReLU, and softmax achieved higher ACC, JAC, and DICE index with low training time than the network without them.

**Table 4:** Performance comparison of the DilatedSkinNet with its modified architectures

Methods	ISIC 2016				ISIC 2017				ISIC 2018			
	ACC	JAC	DICE	Time (s)	ACC	JAC	DICE	Time (s)	ACC	JAC	DICE	Time (s)
DilatedSkinNet	0.940	**0.887**	**0.940**	**10**	0.879	**0.817**	**0.874**	**9**	**0.942**	**0.891**	**0.942**	**14**
Without atrous	0.853	0.735	0.513	12	0.758	0.556	0.460	16	0.825	0.635	0.510	21
Without leakyReLU	0.935	0.855	0.622	11	0.874	0.784	0.578	14	0.863	0.736	0.556	19
Without augmentation	**0.945**	0.884	0.679	11.8	**0.885**	0.788	0.559	12.6	0.942	0.854	0.643	16.5
Sigmoid layer	0.450	0.329	0.496	27	0.465	0.338	0.506	27	0.499	0.331	0.498	51

Time is in seconds on test sets

The higher values are marked in bold

In order to illustrate the generalization of the proposed model, we trained the network on the ISIC 2018 set and evaluated it on the $PH^{2}$ dataset, ISIC 2016, and ISIC 2017 test sets as illustrated in Table 5. The network trained on ISIC 2018 and tested on ISIC 2016 and 2017 test sets showed higher performance with a margin of ($\pm 1\%$) than the network trained individually on ISIC 2016–2018 datasets and evaluated on their respective test sets. For example, the ACC increased from 94.0 to 95.0%, 87.9 to 88.8% on ISIC 2016 and 2017 test sets, respectively. Additionally, a dataset $PH^{2}$ [35] which is widely used in literature studies is included and evaluated using ISIC 2018 trained network.

**Table 5:** Evaluation of DilatedSkinNet trained with ISIC 2018 and tested on $PH^{2}$, ISIC 2016 and 2017 tests sets

Datatset	ACC	JAC	DICE
$PH^{2}$	0.900	0.891	0.708
ISIC 2016 test set	0.950	0.904	0.949
ISIC 2017 test set	0.888	0.818	0.884

**Fig. 4:**
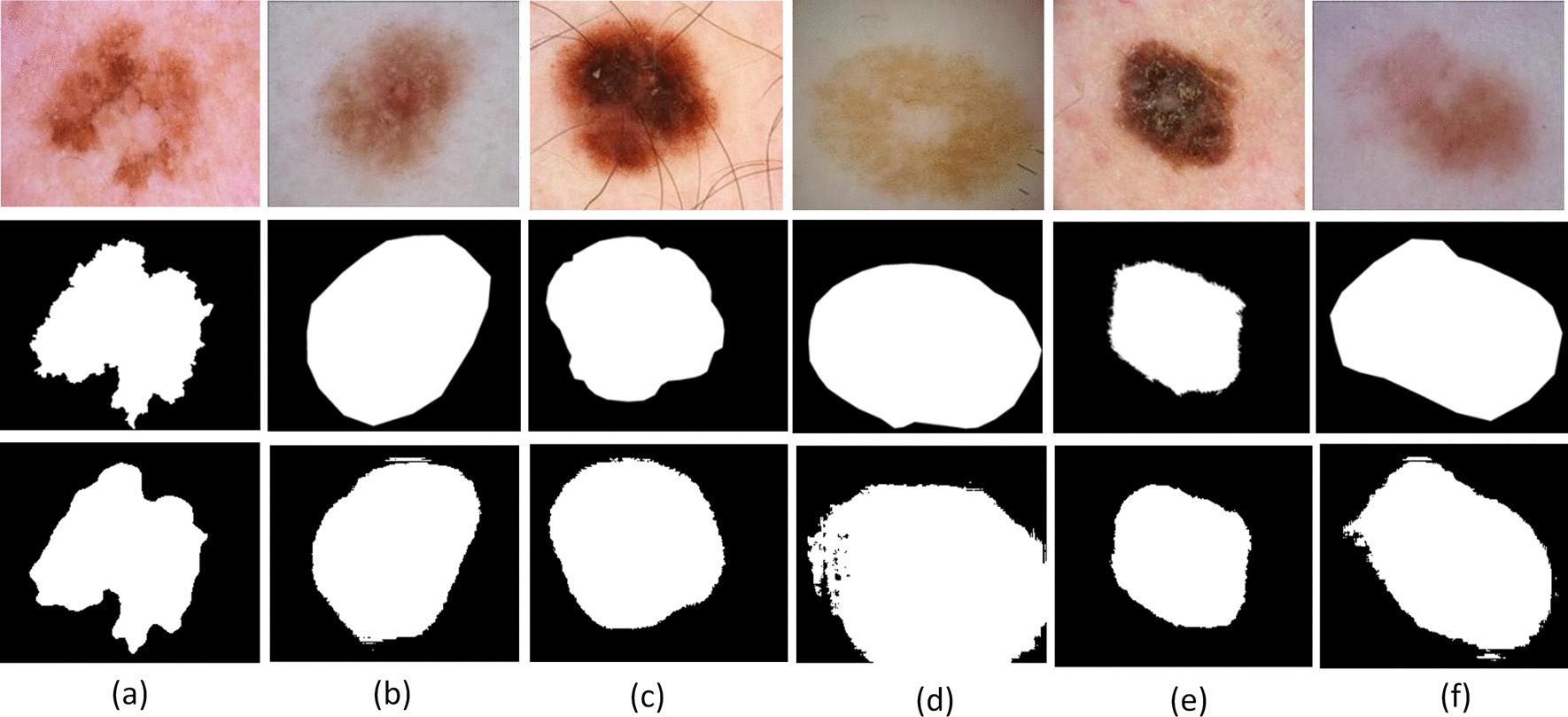
Exemplary pairs of the segmentation result using DilatedSkinNet. The first row shows original images, second row are gold images and the last row are segmented images **a** irregular boundaries, **b** blood vessels, **c** hairlines, **d** color illumination, **e** bubbles, **f** low contrast

The visual outputs predicted by the proposed model for a few samples are shown in Fig. 4 that closely resembles the expected ground truths. Further, in Table 6, the results of the given model are demonstrated in comparison to the existing semantic segmentation networks named UNet, SegNet, and DeepLabv3+. The proposed network showed better performance as compared to the existing segmentation frameworks. The networks were trained by fine-tuning them on the same datasets for conducting a fair comparison. The pixel classification block of these networks was replaced by the new layers segmenting an image into two classes; lesion and background. The same hyperparameter configuration is used, and networks are trained from end to end for training these networks.

**Table 6:** Performance comparison of the DilatedSkinNet with UNet, SegNet, and DeepLabv3+ on ISIC 2016–2018 test sets

Methods	ISIC 2016				ISIC 2017				ISIC 2018			
	ACC	JAC	DICE	Time (s)	ACC	JAC	DICE	Time (s)	ACC	JAC	DICE	Time (s)
U-Net [22]	0.854	0.798	0.832	11	0.764	0.687	0.696	10	0.842	0.793	0.815	24
SegNet [21]	0.908	0.813	0.907	16	0.822	0.679	0.818	16	0.880	0.730	0.879	33
DeepLabv3+ [23]	**0.952**	0.892	**0.952**	16	0.878	0.730	0.881	19	0.939	0.888	0.941	20
DilatedSkinNet	**0.950**	**0.904**	**0.949**	**10**	**0.888**	**0.818**	**0.884**	**9**	**0.942**	**0.891**	**0.942**	**14**

Time is in seconds on test sets

The higher values are marked in bold

**Fig. 5:**
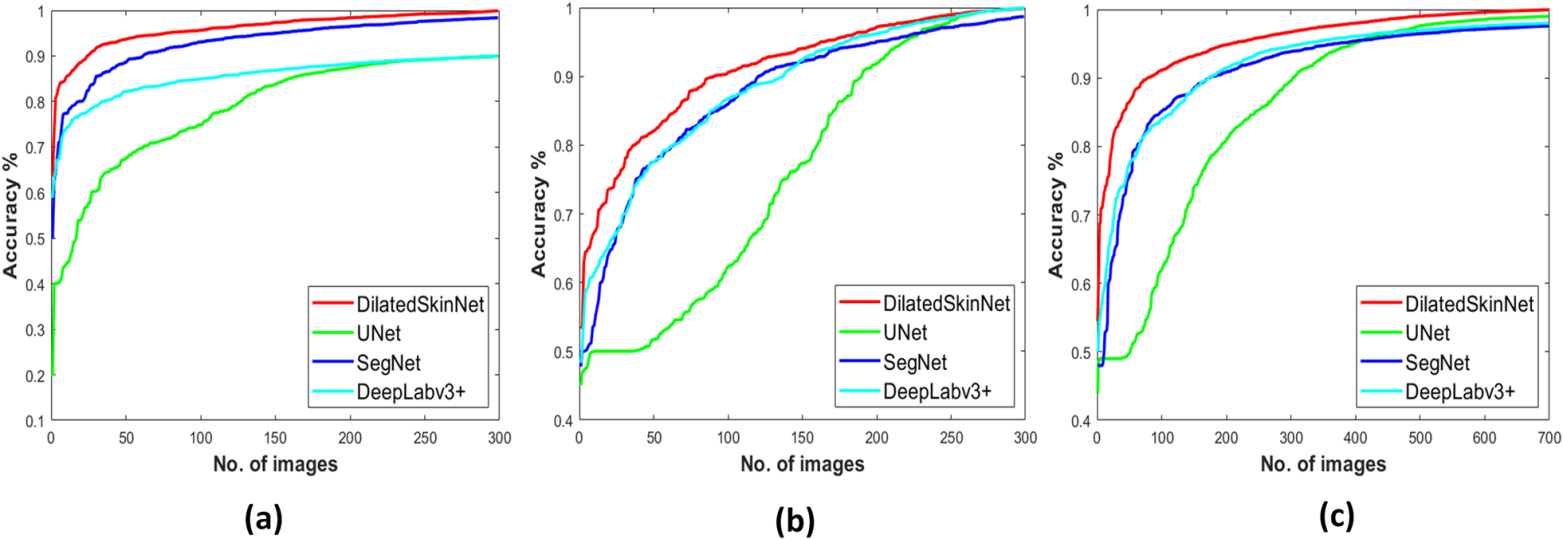
Comparison of networks. Accuracy versus test samples on the ISIC **a** 2016, **b** 2017, **c** 2018 test sets

**Fig. 6:**
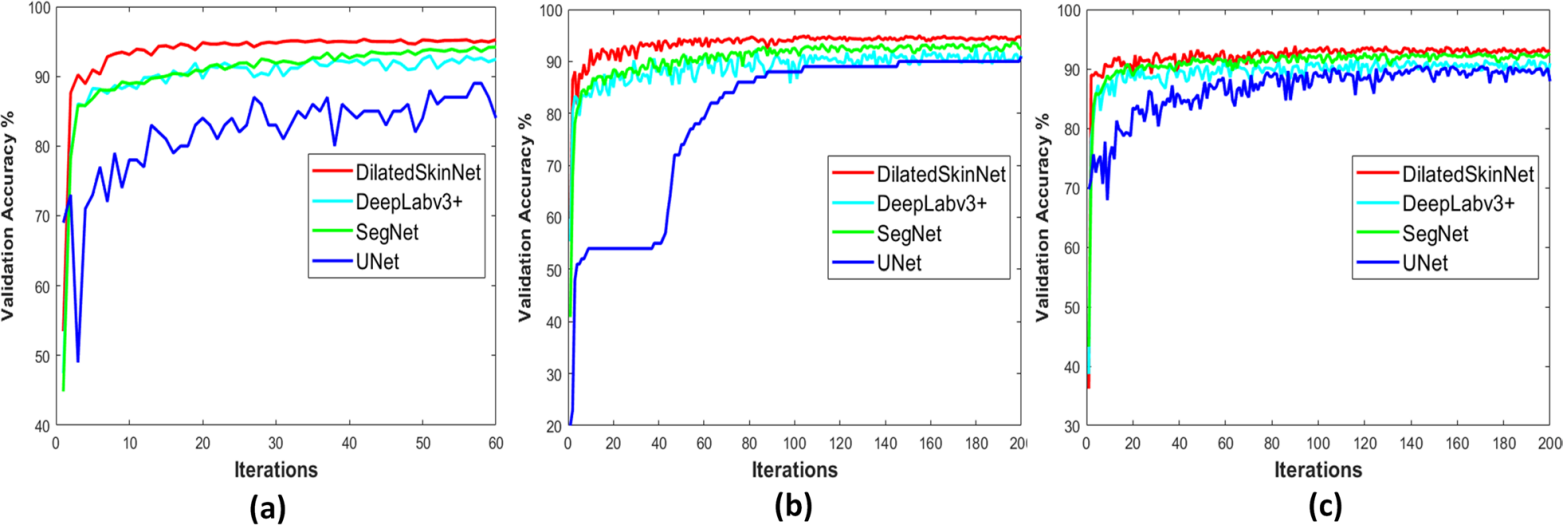
Comparison of networks. Accuracy versus iterations on the ISIC **a** 2016, **b** 2017, **c** 2018 validation sets

**Fig. 7:**
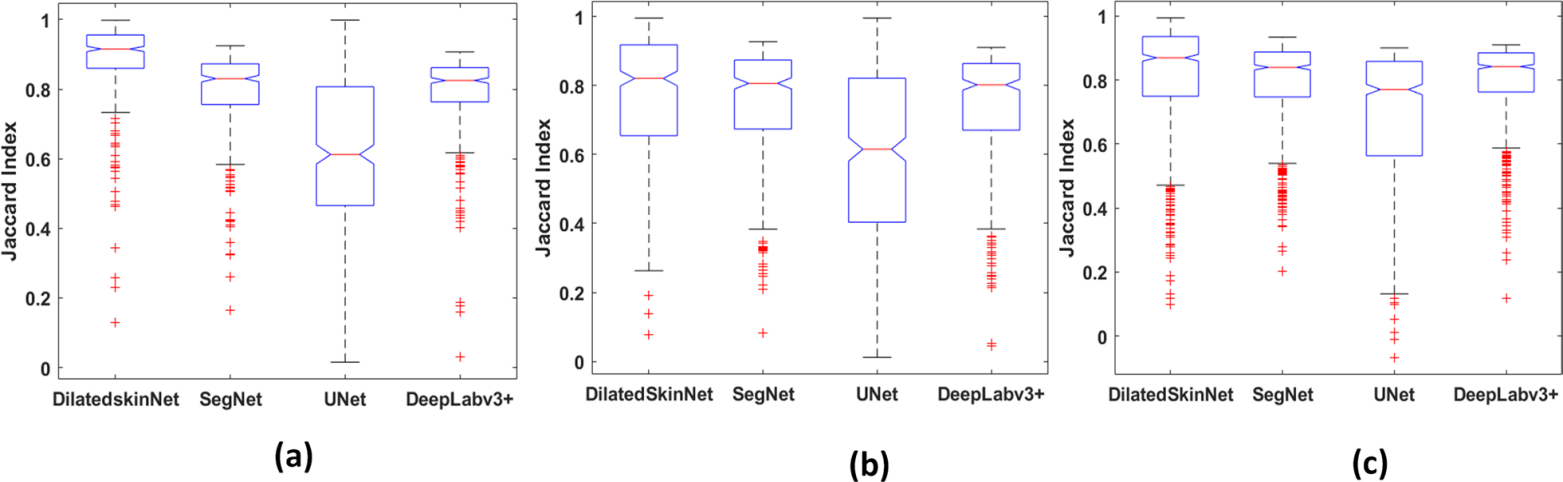
Comparison of networks. Box plots based on Jaccard index of test sets: **a** ISIC 2016, **b** ISIC 2017, **c** ISIC 2018

The performance of these networks is recorded on the individual test sets of three years. The proposed network achieved an average ACC 95.0%, JAC 90.4%, and DICE 94.9% on ISIC 2016, ACC 88.8%, JAC 81.8%, DICE 88.4% on ISIC 2017, and ACC 94.2%, JAC 89.1%, DICE 94.2% on ISIC 2018 dataset. In contrast to this, the JAC index computed by the UNet was 79.8%, 68.7%, and 79.3%, SegNet computed JAC of 81.3%, 67.9%, 73.0%, and DeepLabv3+ gained JAC of 89.2%, 73.0%, and 88.8% on ISIC 2016, 2017 and 2018 datasets respectively. The results show that the proposed network generalized well on the test sets compared to the state-of-the-art semantic segmentation networks. Moreover, the primary advantage of the proposed networks is that it yields high performance in less inference time comparatively. The graphs in Figs. 5 and 6 shows higher accuracy achieved by the DilatedSkinNet on ISIC 2016–2018 test and validation sets, respectively, in comparison to the SegNet, UNet, and DeepLabv3+ networks. Moreover, the box plots in Fig. 7 demonstrate that DilatedSkinNet is efficient in extracting lesion information with a high JAC score as compared to the other models.

To prove the robustness of the network, we compared our model with the top winners of the ISIC challenge 2016–2018, as given in Table 7. The critical parameter used by the ISIC challenge to announce the winner was the JAC index, for which DilatedSkinNet achieved the highest value of 90.4% on ISIC 2016, 81.8% on ISIC 2017, and 89.1% on the ISIC 2018 dataset. Moreover, the last two rows displayed the comparison of DilatedSkinNet with the recent studies published in the years 2019–2021. It is presented that DilatedSkinNet outperformed all given studies by offering higher JAC scores.

**Table 7:** Performance comparison of the DilatedSkinNet with winners of ISIC 2016–2018 challenge and some recent studies

ISIC 2016		ISIC 2017		ISIC 2018	
References	JAC	References	JAC	References	JAC
DilatedSkinNet	**0.904**	DilatedSkinNet	**0.818**	DilatedSkinNet	**0.891**
U. Sanchez [36]	0.843	Yuan [37]	0.765	Qian [38]	0.802
Yu [26]	0.829	Berseth [39]	0.762	Hao [40]	0.799
Rahman [41]	0.822	Bi [42]	0.760	Ji [43]	0.799
Huang [44]	0.811	Menegola [45]	0.754	Yuan [46]	0.798
Xie [47]	0.858	Zafar [48]	0.772	Ali [49]	0.735
Hassan [29]	0.859	Pour [30]	0.782	Lei [50]	0.824
Ashraf [51]	0.859	Liu [33]	0.794	Chu [52]	0.835
Tong [53]	0.845	Ashraf [51]	0.800	–	–
–	–	Tong [53]	0.742	–	–

The experimental results proved that the proposed lightweight network could calculate the expected information efficiently rather than designing a heavy network such as DeepLabV3+. The number of learnable parameters for SegNet, UNet, and DeepLabV3+ is 29M, 31M, 20M, respectively, whereas, for DilatedSkinNet, the number is 3.27K. Additionally, the inferencing time of DilatedSkinNet is also less than others, as shown in Table 6.

**Fig. 8:**
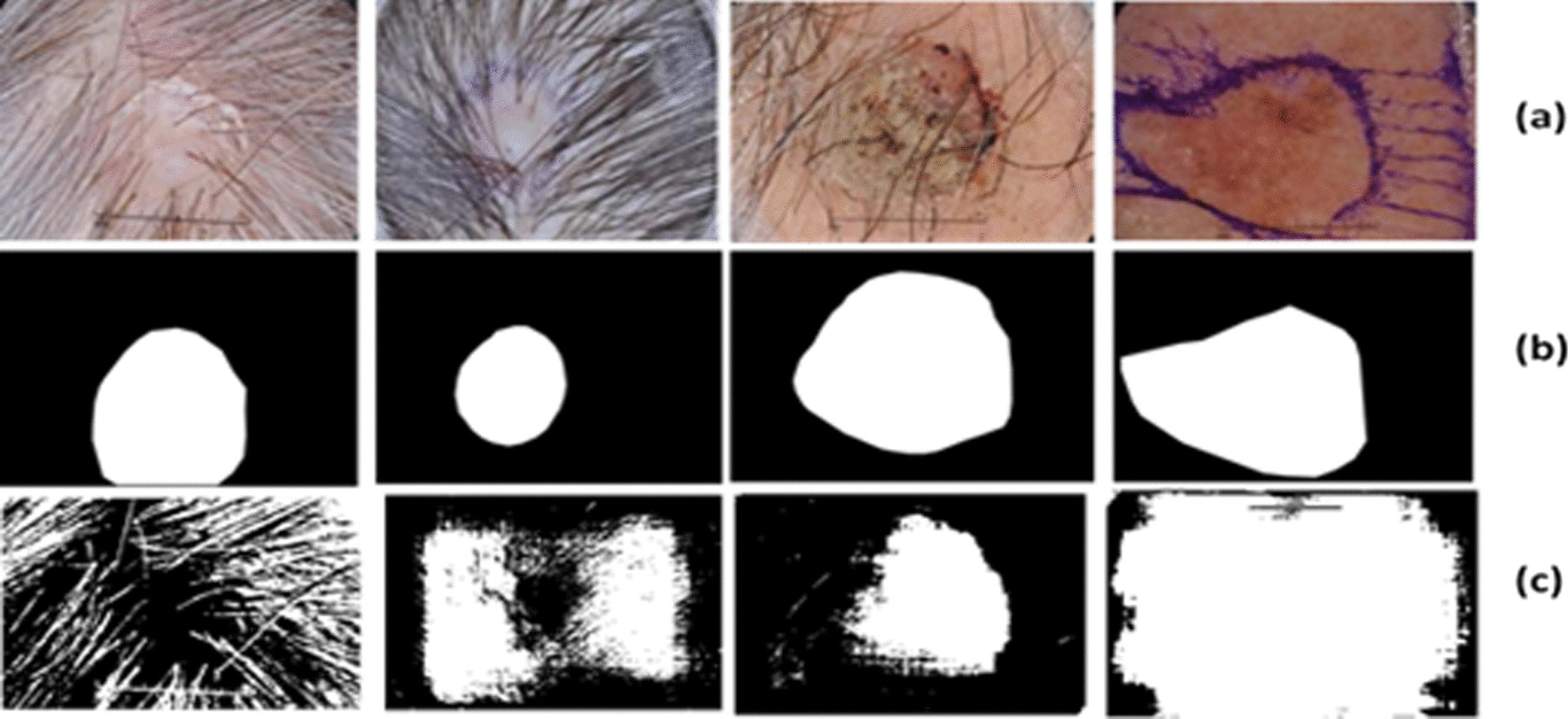
Failure cases. Poor segmentation results using the proposed DilatedSkinNet: **a** Original images, **b** expected segmentation mask, **c** segmented outputs, respectively

Due to the structural dissimilarities, it was challenging to obtain an accurate border of the lesion region. There were some challenging samples, as given in Fig. 8 which were not properly segmented by the given method. The reason for this failure is the presence of noise elements such as dense hairlines and dark ink projections that cause impediments in extracting an accurate region of interest. The network partially segmented these images but was not as accurate as of the ground truth images. Thus, in the future, the design of any pre-processing technique will be taken into consideration for removing noisy elements, primarily hairlines, from images.

## Conclusion

This paper presented a method for the segmentation of lesions in dermoscopic skin cancer images. The proposed network design is a CNN method based on the use of atrous convolutions to replace pooling layers. The atrous dilations can expand the receptive field of the input vector without using pooling layers. These allow each convolution output to contain a wide range of information without extra computations and lose the image’s resolution. The network achieved higher performance by minimizing the cross-entropy loss across mini-batches. The network successfully extracted relevant features from the different dermoscopic skin cancer images and generated segmented image maps. We observed through the experimental results that the proposed network successfully segmented accurate lesion areas that would aid future research work to develop a highly efficient CAD system to classify melanoma and non-melanoma. The network was successful in segmenting the majority of challenging cases, such as irregular boundaries, gel bubbles, low contrast, and color illumination, as given in Fig. 4. However, a few challenging samples, mainly containing dense hairlines, were not accurately segmented illustrated in Fig. 8, which will be considered in the future scope of this research. Based on the higher performance of the DilatedSkinNet, we will focus on its application areas to make it a more general approach, including automatic segmentation and tracking over multiple image sequences.

## Data Availability

The benchmark dataset is used publicly available at (https://challenge.isic-archive.com/data).

## Abbreviations

CNN: Convolutional neural network
ISIC: International skin imaging collaboration
ReLU: Rectified linear unit
BN: Batch normalization
